# An Analysis of Tumor Margin Shrinkage in the Surgical Resection of Squamous Cell Carcinoma of the Oral Cavity

**DOI:** 10.7759/cureus.15329

**Published:** 2021-05-30

**Authors:** Caitlin Burns, Manel Gorina Faz

**Affiliations:** 1 Department of Oral and Maxillofacial Surgery, Hospital Universitari Doctor Josep Trueta, Girona, ESP

**Keywords:** surgical margins, shrinkage, oral squamous cell carcinoma, tumor resection, formalin fixation, oral cavity

## Abstract

Background

Surgical resection of the oral cavity squamous cell carcinoma with clear surgical margins is the key to preventing local recurrence and avoiding the need for adjuvant treatment or margin re-resection. There is often a discrepancy observed between the clinically determined margins of the tumor when it is being resected and the histopathological result after the specimen has been processed.

Methods

A total of six patients who underwent primary surgical resection of oral squamous cell carcinoma between June and October 2020 were included. Surgical margins of the tumor were measured and recorded at three stages of tumor resection: pre-incision, post-resection, and post-formalin fixation. The 1 cm pre-incision anterior margin was compared to both the anterior post-resection and post-formalin fixation margins to document any shrinkage between the different stages of tumor resection.

Results

The overall mean surgical margin shrinkage was 26% (95% confidence interval {CI} 9.34-42.66, p=0.012). The greatest amount of margin shrinkage occurred between pre-incision and post-resection measurements, which is statistically significant at 19.7% (95% CI 7.49-31.83, p=0.009). To a lesser extent, tumor surgical margins also decreased by 12.7% (95% CI -2.66 to 28.09, p=0.083) between post-resection and post-formalin fixation.

Conclusion

﻿Dimensions of tumor surgical margins in oral cavity squamous cell carcinoma specimens decrease from surgical resection to histopathological processing. Most of this shrinkage occurs between the clinically determined pre-incision and immediately after tumor resection in the post-resection measurement. These findings suggest that it might be prudent to consider surgical margin shrinkage when outlining initial margins to ensure adequate and complete resection of the tumor.

## Introduction

The tumors of the oral cavity are those that originate from the lips, gum, tongue, mouth (including the floor and buccal mucosa), retromolar trigon, and hard palate. Of these, more than 90% are squamous cell carcinomas (SCC), and most developed from the floor of the mouth or the ventral aspect of the tongue and floor of the mouth [1,2]. These tumors can develop either ex Novo or from pre-existing dysplastic lesions, the two lesions with the highest risk of developing into cancer being leukoplakia and erythroplakia [3,4].

The basis of the treatment in any tumor (T) stage is primary resection of the tumor whenever possible and follow-up attitude will depend on the outcome of this surgery. In cases where the pathology report comes back with adverse features (positive margins, close margins, extranodal extension, pT3 or pT4 primary, pN2 or pN3 nodal disease, nodal disease in levels IV or V, perineural invasion, vascular invasion or lymphatic invasion), first-line follow up treatment is re-resection of the tumor bed. In the event that follow-up surgery is not feasible or unsuccessful, adjuvant treatment with radiotherapy/chemotherapy is indicated [5]. The only feature that is in the clinician’s control so as not to obtain adverse features from primary surgery is obtaining clear margins when resecting the tumor. Clear margins are defined as a distance of 5 mm or more from the invasive tumor to the resected margin as determined by histological confirmation [5]. Tumor-free margins are an essential aspect in decreasing the risk for local recurrence [6]. Positive or close margins have been demonstrated to increase relapse and are one of the indicators for adjuvant treatment after primary tumor resection. Therefore, an effort should be made to spare the most patients possible of the burden of undergoing a second surgery or radiotherapy/chemotherapy by obtaining clear surgical margins. National Comprehensive Cancer Network (NCCN) guidelines consider adequate surgical resection to be 1.0-1.5 cm of visible and palpable mucosa when resecting the tumor [5].

The Department of Oral and Maxillofacial Surgery of Hospital Universitari Dr. Josep Trueta, Girona, Spain, has observed a discrepancy between the clinically determined margins of the tumor when it is being resected and the histopathological result given after the specimen has been analyzed. This anecdotal evidence suggests that surgical margins shrink to less than 1 cm after histopathological processing. This observation is supported by a few studies with small sample sizes which concluded that there is a significant overall margin shrinkage of 11-59.02%, but consensus has not been reached as to the percentage of overall shrinkage nor in which stage of tumor resection is the greatest [7-11]. The main objectives of this study, therefore, include determining the overall margin shrinkage, as well as in which stage there is the greatest amount of shrinkage. This would perhaps open up a space for a discussion into a possible revision of the preferred dimensions of surgical margins in primary resection of squamous cell carcinoma of the oral cavity, which would take into account the phenomenon of margin shrinkage that occurs post tumor resection and histopathological processing.

## Materials and methods

All patients were informed before their participation in the study and their written consent was obtained by them signing the informed consent sheet provided. The study received approval from the Ethics Committee of Hospital Universitari Dr. Josep Trueta.

Study design and participants

This prospective observational study took place in the Department of Oral and Maxillofacial Surgery in Hospital Universitari Dr. Josep Trueta, Girona, Spain, between June and October 2020. During this time, all patients undergoing primary surgical resection of the tumor with curative intent for SCC of the oral cavity were included if they met inclusion criteria and did not have any exclusion criteria. Inclusion criteria were biopsy-confirmed oral SCC with no previous treatment of the oral cavity, either presence or absence of human papillomavirus (HPV) infection, and good general condition to withstand a major surgical procedure. Exclusion criteria were previous major oral surgery, history of radiation of the oral cavity, patients with loco-regional recurrence or distant metastases, and connective tissue disease.

A total of seven patients were considered to be included in this study; one was excluded because of the previous radiotherapy of the oral cavity. All six patients were males and ranged from 51 to 91 years (mean age 65.5 years), one tumor was obtained from the buccal mucosa (16.6%) and the other five from the tongue (83.3%).

Variables

Surgical margin measurements were measured in the three different stages of tumor resection (pre-incision, post-resection, and post-formalin fixation). The percentage of margin shrinkage that occurred between each stage of tumor resection as well as overall shrinkage was also quantified. Additionally, the number of patients who needed adjuvant therapy attributable to the margin shrinkage in the early-stage tumors (T1N0, T2N0, T2N1, T2N1) was detected.

Other covariables considered were patient demographics (age and sex), tumor anatomical site, exposure to risk factors such as smoking and alcohol consumption, cTNM (clinical tumor, node, metastasis), and pTNM (pathological tumor, node, metastasis) as determined by the NCCN guidelines and tumor grade. The variables and covariables together with their measurement unit can be seen in Table 1.

**Table 1:** Variables, Covariables, and Their Respective Measurement Units Mm: millimeters; TNM: tumor, nodes, and metastases; G1: grade 1; G2: grade 2; G3: grade 3

Variable	Measurement unit
Surgical margin measurement	mm
Percentage of shrinkage between stages	%
Need for adjuvant therapy	Yes/No
Age	Years
Sex	Male/Female
Tumor anatomical site	Tongue/Buccal mucosa
Smoking	Yes/No
Alcohol consumption	Yes/No
cTNM	TNM staging
pTNM	TNM staging
Tumor grade	G1/G2/G3

Data collection

Pre-incision margins were determined by inspection and palpation by the attending surgeon, then the borders of the tumor were then outlined with a sterile marker. A sterile ruler was used to measure 1 cm out from the previously outlined border of the tumor in the anterior, posterior, superior, and inferior aspects of the tumor and any other area deemed necessary, the dots were connected to form the 1 cm circumferential surgical margin. The anterior margin was marked with a suture to be used as a reference point for histopathological examination.

Post-resection margins were measured with the same ruler as the pre-incision margins shortly after the tumor was resected either by the main investigator or another member of the surgical team. The anterior margin was used from this point on as a reference for all tumor specimens.

**Figure 1:**
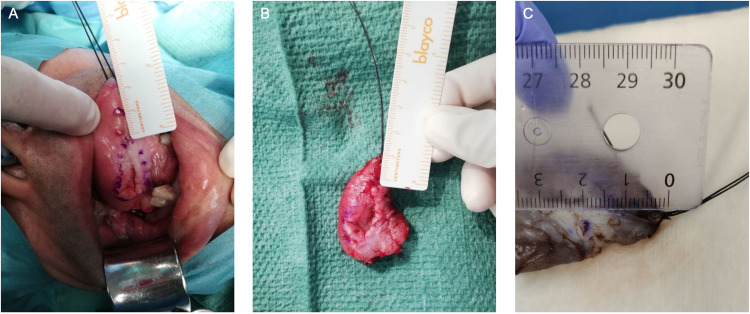
Images of The Three Stages of Tumor Resection (A) Pre-incision, (B) post-resection, and (C) post-formalin fixation. Surgical margins measured in centimeters. Tumor located on the right lateral inferior border of the tongue.

Post-formalin margins were measured one to two days after the surgery in the pathology department with a ruler when the tumor had undergone histopathological processing but before the lamination process. The pictures were taken of the in-situ, post-resection, and post-formalin fixation measurement process, an example of which can be seen in Figure 1.

Statistical analysis

Quantitative patient demographic data is expressed as a mean with its standard deviation and qualitative demographic data is expressed as a proportion with its 95% confidence interval to estimate the population proportion. Statistical analysis was conducted using IBM SPSS Statistics for Macintosh Version 25.0 (Armonk, NY: IBM Corp.). Surgical margin shrinkage was determined through post hoc one-way analysis of variance testing (ANOVA). The percentage of variation between the first two groups (pre-incision and post-resection) and the latter two groups (post-resection and post-formalin fixation), as well as overall surgical margin shrinkage, was determined through post hoc ANOVA and linear regression analysis. The p-value of ≤ 0.05 was considered statistically significant.

## Results

A total of six patients were included in the study, of these, all were men and ages ranged from 51 to 91 years (mean age 65.5 years). All tumors were obtained from the oral cavity, one from the buccal mucosa (16.6%) and the other five from the tongue (83.3%), all six were histologically confirmed SCC. Tumor staging was as follows: four pT1N0M0, one pT2N2aM0, and one pT3N0M0. Patients’ demographic characteristics are summarized in Table 2.

**Table 2:** Patients’ Demographic Data SD: standard deviation; CI: confidence interval

Studied variable	n	Estimator (SD for mean and 95% CI for proportions)
Age (years)	6	65.5 (50-81)
Total patients		
Male	6	100% (95% CI 60.9-100)
Female	0	0% (95% CI 0-39.0)
Tumor site		
Tongue	5	83.3% (95% CI 43.6-96.9)
Buccal mucosa	1	16.6% (95% CI 3.0-56.3)
Tumor stage (pT)		
T1	4	66.6% (95% CI 30.0-90.3)
T2	1	16.6% (95% CI 3.0-56.3)
T3	1	16.6% (95% CI 3.0-56.3)
Nodal stage (pN)		
N0	5	83.3% (95% CI 43.6-96.9)
N1	0	0% (95% CI 0-39.0)
N2	1	16.6% (95% CI 3.0-56.3)
Exposure to risk factors		
Smoking	6	100% (95% CI 60.9-100)
Smoking + alcohol consumption	4	66.6% (95% CI 30.0-90.3)

Of the six examined patients, three had clear margins in the final histopathological result, two were close, and one was positive. Three measurements were obtained from each specimen, first, the pre-incision surgical margin measurement, which was always 10 mm as determined by the NCCN guidelines, second, the post-resection margin measurement after the tumor had been resected, and lastly, the post-formalin fixed margin once the specimen had undergone histopathological processing. Of the six examined specimens, one measurement in the post-formalin fixation group was unable to be measured before the specimen was laminated for histological examination. Consequently, there were six pre-incision and post-resection measurements and five post-formalin fixation measurements.

**Figure 2:**
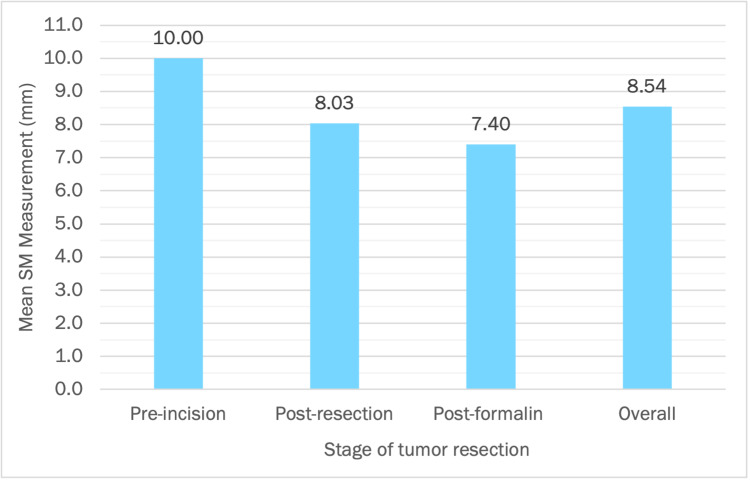
Mean Margin Measurements (in mm) in the Different Stages of Tumor Resection Mean margin measurements were as follows: pre-incision 10 mm (95% CI 10-10), post-resection 8.03 mm (95% CI 6.81-9.25), and post-formalin fixation 7.40 mm (95% CI 5.73-9.06). The number of samples was six specimens for both pre-incision and post-resection measurements and five for post-formalin fixation measurement. The difference between the mean measurements in the different groups was statistically significant (p=0.002). SM: surgical margin

Mean margin measurements were as follows: pre-incision 10 mm (95% confidence interval {CI} 10-10), post-resection 8.03 mm (95% CI 6.81-9.25), and post-formalin fixation 7.40 mm (95% CI 5.73-9.06), which can be seen in Figure 2.

**Figure 3:**
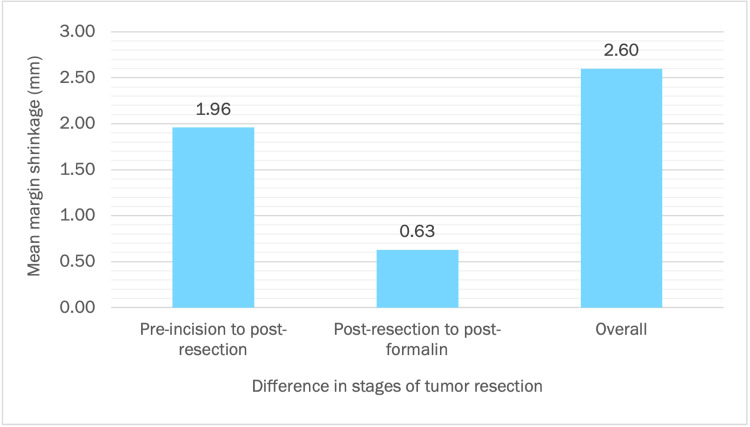
Mean Margin Shrinkage (in mm) Between the Different Stages of Tumor Resection Overall surgical margin shrinkage was 2.60 mm (95% CI 1.01-4.18, p=0.011). Shrinkage from pre-incision to post-resection was 1.96 mm (95% CI 0.45-3.47, p=0.011) and from post-resection to post-formalin was 0.63 (95% CI -0.94 to 2.21, p=0.56). Statistical significance is denoted with an asterisk (*).

Overall surgical margin shrinkage was 2.60 mm (95% CI 1.01-4.18, p=0.011). Shrinkage from pre-incision to post-resection was 1.96 mm (95% CI 0.45-3.47, p=0.011) and from post-resection to post-formalin was 0.63 (95% CI -0.94 to 2.21, p=0.56) (Figure 3).

Therefore, according to the data collected, from pre-incision to post-resection measurement, there was a 19.7% (95% CI 7.49-31.83, p=0.009) reduction of margins, from post-resection to post-formalin fixation, a 12.7% (95% CI -2.66-28.09, p=0.083) reduction and overall a 26% (9.34-42.66, p=0.012) margin reduction.

Of the six patients, three received adjuvant therapy, two were as a result of close or positive margins, and the remaining one was due to a positive adjacent margin of the posterior tumor bed but with clear surgical margins, indicating the presence of a second tumor posterior to the one originally resected. The two patients who received adjuvant treatment due to close or positive surgical margins were treated with re-resection of the tumor bed following the recommendations of the NCCN guidelines.

By taking into account, the overall shrinkage was 2.60 mm, only patients who have pathology reports with tumor margins between 2.4 mm and 5 mm would benefit from wider surgical margins so as not to require adjuvant treatment due to close margins (under 5 mm as defined by the NCCN guidelines).

## Discussion

A discrepancy between the clinically determined margins intraoperatively and the histopathological results after tumor specimen processing has been observed in tumors of the oral cavity. Obtaining clear surgical margins is critical in decreasing the risk of local recurrence and the need for adjuvant treatment or margin re-resection after primary surgical resection. Surgical margin shrinkage could lead to seemingly smaller margins on the pathology report, which in turn could lead to more patients receiving adjuvant treatment or a second surgery for margin re-resection.

In this study, surgical margins were measured macroscopically in three stages of tumor resection: pre-incision, immediately post-resection, and post-formalin fixation, to quantify the shrinkage of surgical margins of SCC of the oral cavity in patients undergoing primary surgical resection of the tumor with curative intent. Additionally, the stage in which there was the greatest amount of shrinkage was determined.

This study concluded that there is an overall statistically significant 26% (95% CI 9.34-42.66, p=0.012) surgical margin shrinkage. This is in line with five previous studies which found similar reductions in surgical margins of oral cavity tumors. Mistry et al. found an overall shrinkage from pre-incision margins to post-resection margins of 22.7% [7]. Cheng et al. observed ﻿a 59.02% reduction in surgical margins of any oral cavity location, specifically a 71.9% reduction in the buccal mucosa, mandibular alveolar ridge, and retromolar trigone; and a 42.14% reduction in the tongue [8]. El-Fol et al. found that the mean discrepancy between the pre-incision margins and the histopathological margins of all close and positive margins was 47.6% for the buccal mucosa and 33.3% for the tongue [9]. Mohiyuddin et al. ﻿identified a reduction of almost 25% from the closest histopathologic margin compared with the pre-incision margin [10]. Lastly, U﻿mstattd et al. concluded that the overall mean shrinkage of tumor size was 10.7%, and mean margin shrinkage was 11.3% [11]. No studies have been done that have found no significant decrease in surgical margins. However, in the studies mentioned previously, there is no consensus regarding the amount of shrinkage that surgical margins undergo after tumor processing. Surgical margin shrinkage in this study was found to be 26%, which is consistent with these previous findings which ranged from 11.3% to 59.02%.

We found that the greatest amount of surgical margin shrinkage is from pre-incision measurement to post-resection measurement, which was found to be statistically significant at 19.7% (95% CI 7.49-31.83, p=0.009). Shrinkage from post-resection to post-formalin fixation was found to be less at 12.7% (95% CI -2.66 to 28.09, p=0.083) but was not statistically significant. This suggests that the majority of shrinkage is due to intrinsic tissue properties rather than the effects of extrinsic tissue processing [12-14]. Only one other study has been performed that has examined in which of the stages of tumor processing there is the greatest reduction in surgical margins. Umstattd et al. found there was a statistically significant 14.9% shrinkage occurring pre-resection to post-resection and 3.7% shrinkage occurring post-resection to the post-formalin fixation that was not statistically significant [11].

It is indicated in patients with close or positive surgical margins on the histopathological report after primary tumor resection that either margin re-excision or adjuvant treatment should be performed, pending the collective decision of the Head and Neck Tumor Committee [5]. It is therefore in the surgeon’s control to avoid the need for the patient to be exposed to the added morbidity of receiving adjuvant radiotherapy/chemotherapy or a second surgical procedure to re-excise additional surgical margins in cases of the early-stage tumors (T1N0, T2N0, T2N1, T2N1) without other adverse features [15]. This study aimed to discover how many patients would undergo additional adjuvant treatment due to close or positive surgical margins that could be attributed to the observed surgical margin shrinkage that occurs after the specimen has been resected. As stated previously, an overall margin shrinkage of 2.60 mm was found, and close margins are defined as those under 5 mm. Therefore, patients who have received histopathological reports indicating margins between 2.4 mm and 5 mm would be those affected by this phenomenon. Of the patients examined in this study, none fit this description, so the proportion of patients who would be affected by the surgical margin shrinkage could not be determined. No previous studies have been performed in this line of research due to the fact that surgical margin shrinkage has not yet been established as a factor affecting the need for adjuvant treatment after primary surgical resection of the tumor. Therefore, further investigation needs to be done comparing the standard 1 cm surgical margins to expanded surgical margins that would take shrinkage into account to expose any differences in the number of patients who then require margin re-resection or adjuvant treatment due to close or positive histopathological margins. The morbidity of increased margin size would also need to be compared to the morbidity of adjuvant radiotherapy/chemotherapy or a second surgical procedure to re-excise margins from the tumor bed to determine whether larger initial surgical margins are preferable to potential later margin re-resection or adjuvant treatment.

The main limitation of this study was the small sample population that was included due to the reduced time frame in which this study took place. A multicentric study to replicate the results would be necessary to validate these findings, in which a larger sample size would increase the power and therefore increase the validity of the hypothesis that there is a shrinkage between the clinically examined pre-incision margins previous to surgical resection of oral SCC and the margins measured after histopathological tumor processing. It would also be prudent to take into consideration the different anatomical locations of tumors in the oral cavity since previous studies have observed different margin discrepancies according to tumor site [7-9,11]. Though surgical margin shrinkage has been theorized as a cause for close or positive histopathological margins, occult infiltration should not be dismissed as a cause for the observed margin discrepancy.

Another factor that could be taken into account to determine if it has any effect on tumor margin shrinkage is the instrument used for tumor resection [16]. In this study, a harmonic scalpel was used in all five glossectomies and a monopolar cautery in the resection of the buccal mucosa. Accepted instruments for resection include a scalpel, monopolar cautery, or harmonic scalpel, therefore it could be relevant to investigate if the instrument used has an impact on tumor shrinkage due to varying tissue contraction or thermal damage.

Though it would be compelling to suggest that larger surgical margins should be adopted when treating oral cavity carcinomas with primary surgical resection, currently no study has been performed relating surgical margins over 1 cm with an improved prognosis in these types of tumors.

## Conclusions

Significant shrinkage of surgical margins occurs following resection of squamous cell carcinomas of the oral cavity, an overall 26% margin shrinkage can be expected after resection and histopathological processing from the time of initial resection for oral cavity tumors. Most of this shrinkage occurs between pre-incision and post-resection, and to a lesser extent between post-resection and post-formalin fixation. The proportion of patients that would receive adjuvant treatment following primary resection of the tumor due to margin shrinkage could not be determined in this study. These findings suggest that this phenomenon of margin shrinkage could be considered when outlining the initial surgical margins to ensure the adequacy of complete resection and avoid adjuvant therapy due to close or positive margins.
